# Serum Autofluorescence and Biochemical Markers in Athlete's Response to Strength Effort in Normobaric Hypoxia: A Preliminary Study

**DOI:** 10.1155/2019/5201351

**Published:** 2019-12-06

**Authors:** Zofia Drzazga, Izabela Schisler, Stanisław Poprzęcki, Anna Michnik, Miłosz Czuba

**Affiliations:** ^1^Faculty of Science and Technology, University of Sillesia in Katowice, Chorzów 41-500, Poland; ^2^Department of Physiological and Medical Sciences, Department of Biochemistry, The Jerzy Kukuczka Academy of Physical Education, Katowice 40-065, Poland; ^3^Department of Kinesiology, Institute of Sport, Warsaw 01-982, Poland

## Abstract

The human organism has the ability to adapt to hypoxia conditions. Training in hypoxia is used in sport to improve the efficiency of athletes; however, type of training affects the direction and scope of this process. Therefore, in this study, the usefulness of serum fluorescence spectroscopy to study the assessment of athlete's response to strength effort in hypoxia is considered in comparison with biochemical assay. Six resistance-trained male subjects took part in a research experiment. They performed barbell squats in simulated normobaric hypoxic conditions with deficiency of oxygen 11.3%, 13% 14.3% compared to 21% in normoxic conditions. Fluorescence intensity of tyrosine revealed high sensitivity on strength effort whereas tryptophan was more dependent on high altitude. Changes in emission in the visible region are associated with altering cell metabolism dependent on high altitude as well as strength training and endurance training. Significant changes in serum fluorescence intensity with relatively weak modifications in biochemical assay at 3000 m above sea level (ASL) were observed. Training at 5000 m ASL caused changes in fluorescence parameters towards the normobaric specific values, and pronounced decreases of lactate level and kinase creatine activity were observed. Such modifications of fluorescence and biochemical assay indicate increased adaptation of the organism to effort in oxygen-deficient conditions at 5000 m ASL, unlike 3000 m ASL. Fluorescence spectroscopy study of serum accompanied by biochemical assay can contribute to the understanding of metabolic regulation and the physiological response to hypoxia. The results of serum autofluorescence during various concepts of altitude training may be a useful method to analyze individual response to acute and chronic hypoxia. An endogenous tryptophan could be exploited as intrinsic biomarker in autofluorescence studies. However, these issues require further research.

## 1. Introduction

Hypoxia is a state when tissue oxygenation in certain body organs or the whole body is insufficient compared to oxygen demand. The physiological cause of hypoxia in the organism is its exposure to natural (hypobaric hypoxia) or simulated (normobaric hypoxia) altitude. Except for static exercise and short-term high-intensity exercise [1, 2], hypoxia contributes to reduced exercise capacity [3, 4].

In high-altitude conditions, the human body is exposed to a disorder syndrome called acute mountain sickness (AMS) even at a level critical for survival [5]. Respiratory, cardiovascular, muscular, hormonal, and metabolic adjustments have been observed [6]. On the other hand, many people live in the high mountains, which proves the great adaptability of the human organism. The physiological and morphological effects of high altitude in humans are the subject of studies by many researchers [7]. Based on the pattern of adaptive changes occurring in the human body in response to hypoxia, it can be concluded that the key factors for increased exercise capacity in hypoxia are ventilatory adaptation, improvement of the process of oxygen supply in tissues resulting from lowering hemoglobin (Hb)-O_2_ affinity, and improvement of blood oxygen-carrying capacity [8–10]. It is worth noting that erythropoiesis-regulated increase of blood oxygen-carrying capacity is a slow response and constitutes a long-term adaptation to hypoxia [11, 12].

Additionally, significant changes in the level of proteins were found in the plasma of people living in the high mountains, especially of the low-abundance proteins [13, 14]. Human plasma proteome analysis [13] identified, e.g., upregulated proteins such as vitamin D-binding proteins, hemopexin, alpha-1 antitrypsin, haptoglobin, apolipoprotein A, transthyretin, and hemoglobin beta chain, whereas the downregulated proteins were transferrin, serum amyloid, and component C3 and C4A. These inflammation inhibitors are thought to play a positive anti-inflammatory role in high altitude.

One of the better-known adaptive responses to hypoxia is the activation of the HIF-1 transcription factor, which is a master regulator of the cellular and systemic homeostatic response to hypoxia by activating transcription of many genes, including those involved in, e.g., energy metabolism [15]. HIF-1 undergoes conformational changes in response to changes in oxygen concentration to induce an increase in oxygen availability by regulating erythropoiesis and angiogenesis [16]. An increase in the amount of erythrocytes has been proven clearly. The hematological and morphological adaptations lead to the improvement of energy metabolism and allow for efficient functioning of the human body in hypoxic conditions. Therefore, e.g., soldiers' acclimatization to altitude before their deployment in the high mountains was applied [17].

Knowledge about physiological and morphometric changes induced by hypoxia is also used in sport [18]. It is known that skeletal muscle is one of the most sensitive tissues for oxygen, due to its different levels during muscle contraction, especially during physical exercise. Altitude exposure increases erythropoiesis leading to more red blood cells and increases hemoglobin mass, facilitating the transport of oxygen to tissues [19]. Muscle hypertrophy induced by resistance training in hypoxia was reported [20, 21].

However, the regulation of muscle mass by environmental hypoxia and protein metabolism in human skeletal muscle is complex [22]. Generally, hypoxic training is considered an effective method to improve the efficiency of sportsmen in different sport disciplines although some researchers have stated the lack of additional benefits from training in hypoxia for athletes [23]. Effects of training in hypoxic conditions depend on many different factors, e.g., high altitude, long- or short-term training, diet, and kind of exercise being performed.

In the intermittent hypoxic training method, athletes living in normoxic conditions train in natural or simulated hypoxic conditions. Improvements in aerobic capacity and endurance performance caused more by muscular and systemic adaptations than hematological adaptive mechanisms to hypoxia were reported [13, 15, 17]. Increased fiber cross-sectional area and skeletal muscle mitochondrial density and elevated capillary-to-fiber ratio were observed [22, 24] due to IHT. Recently, a possibility of an increase in the concentration of acute-phase haptoglobin and/or C-reactive proteins in the serum of men training in normobaric hypoxia was suggested by calorimetric measurements [25].

Effects of training in hypoxic conditions are usually analyzed in the framework of physiological and biochemical parameters of athletes but not by fluorescence spectroscopy of serum, which was applied in our studies. Fluorescence spectroscopy as a sensitive and noninvasive method, which measures the emission intensity of the sample at very low concentration of fluorophores in biological systems [26], plays an important role in the medical diagnosis of cancer [27–31] as well as nononcological states [32–34]. It is worth recalling that the development of endogenous and exogenous fluorescence studies in metabolic disorders and in particular the presence of neoplasia has led to the currently used photodynamic and diagnosis (PDD) and photodynamic therapy (PDT) [35]. There are also reports demonstrating that fluorescence studies of serum can be potentially useful in distinguishing deviations from the normal status of serum in various types of disease [36–38]. Recently, the impact of endurance training and testosterone treatment on rat blood serum using fluorescence spectroscopy has been determined [39].

Measurement of autofluorescence is not a conventional test used to monitor the athlete's training status. Nevertheless, in our opinion, it can refine the results of exercise physiological and biochemical markers. For that reason, the aim of this study was to assess blood sera of athletes subjected to strength exercise in normobaric hypoxic conditions using fluorescence spectroscopy and biochemical assay. Exercise-induced muscle damage in sports medicine is routinely monitored by biochemical markers such as creatine kinase (CK) and lactate dehydrogenase (LDH) activities [40–42] and lactate level due to training. The question is whether autofluorescence of blood serum provides new information and to what degree it is compatible with biochemical markers routinely used in the estimation of efficiency of sportsmen during intermittent hypoxic training. Therefore, we suppose that exposure to acute hypoxia in combination with resistance exercise can affect both spectral fluorescence parameters of serum and biochemical markers of muscle damage. We hypothesized that autofluorescence of blood serum could complement the measurement of typical biochemical markers routinely used in training load assessment during strength training in normoxia and hypoxia. To test these working hypotheses, we measured autofluorescence of blood serum and activities of creatine kinase (CK), lactate dehydrogenase (LDH), and concentration of lactate (LA) during strength effort in acute normobaric hypoxic conditions.

## 2. Materials and Methods

### 2.1. Participants

Ten healthy, physically active male volunteers with at least 5 years of training experience were chosen by trainers of the Academy of Physical Education in Katowice to participate in normobaric hypoxia experiment. All participants declared that they had not been in the last year at an altitude of 2000 m above sea level. For seven days before and during the experiment, the subjects were on a standardized diet with 50–60% carbohydrate, 15–20% protein, and 20–30% fat. They were also obliged to abstain from strenuous exercise three days before each testing session. No dietary supplements for athletes were permitted during the 3 weeks before and during the experiment. All participants possessed a current medical examination, without any contraindications to performing exhaustive exercise in both normoxia and a hypoxic environment. Athletes were informed about the purpose and the nature of the research before giving their written consent to participate in the experiment. The studies were performed in accordance with the ethical standards of the responsible committee on human experimentation (institutional and national) and with the Helsinki Declaration. The study protocol was approved by the Ethics Committee of the Jerzy Kukuczka Academy of Physical Education in Katowice (Certificate of approval no. 4/2011). However, some subjects did not complete the exercises at all altitudes. In results, the study included 6 resistance-trained male subjects (mean ± SD: age 24.1 ± 1.0 year, body mass 82.9 ± 15.2 kg, body height 175.5 ± 7.5 cm, fat content 11.3 ± 4.6%, and BMI 25.6 ± 3.8 kg·m^−2^).

### 2.2. Experimental Design

Initially, the one repetition maximum (1RM) test protocol was measured in normoxic and hypoxic (3000 m, 4000 m, and 5000 m above the sea level) conditions using the Myotest device with the Half-Squat Profile test and the equation 1RM = load *x* (1 + 0.0033), where *x* = performed number of repetitions [43].

The experiment comprised four stages with seven-day intervals. The participants of our experiment performed squats with a barbell with individual weight (70% 1RM) in each environmental condition. They performed 10 sets of 12 repetitions with 5 min of rest periods between sets. Participants entered the exercise test after a 24-hour rest period.

The exercise test was carried out in normoxic conditions, corresponding to the level of the sea and fraction of inspired oxygen 21% (average work in the session 5.17 kJ), and normobaric hypoxic conditions: 3000 m ASL where the oxygen content was 14.3% (average work 5.23 kJ), 4000 m ASL where the oxygen content was 13% (average work 5.04 kJ), and 5000 m ASL where the oxygen content was 11.3% (average work 5.21 kJ). All tests were performed in a normobaric hypoxia chamber with the climate system LOS-HYP_1/3 NU (LOXYS SYSTEMS, Germany).

### 2.3. Blood Serum Assessment

Blood serum was analyzed using biochemical assay and fluorescence spectroscopy. Blood samples were drawn at about 1 min before the exercise, 3 min after the exercise in the normobaric hypoxia chamber, and then after 1 h and 24 h of restitution. Serum was obtained by centrifugation at 3500 rpm for 10 min (4°C). Fresh plasma samples were assayed for activities of creatine kinase (CK, EC 2.7.3.2) and lactate dehydrogenase (LDH, EC 1.1.1.27), and concentration of lactate (LA), using diagnostic kits from Randox Laboratories (CK522, LD3818, and LC2389, respectively). Serum samples were stored at −20°C before the fluorescence study.

The fluorescence study was carried out for undiluted and diluted serum samples with redistilled water (1 : 20). The pH value of the undiluted samples was in the range of 8.6–8.8 and that of the diluted samples was in the range of 6.5–7.0. Complete excitation-emission matrices were registered for serum samples: before (A) and about 3 minutes after exercise (B) and then after 1 hour (C) and 24 h (D) of restitution using a Hitachi F-2500 spectrophotometer with FL Solutions software.

Control group in our studies were elite sportsmen, who performed endurance exercise in normoxia conditions. Their blood serum fluorescence and biochemically determined results were taken as the reference level. Data from completed measurements were analyzed statistically (*n* = 6).

### 2.4. Statistical Analysis

The statistical analysis with Statistica 13 was performed on both fluorescence and biochemical variables obtained for serum. The assumptions for the use of parametric tests were checked using Shapiro–Wilk and Levene tests to evaluate the normality of the distributions and the homogeneity of variance, respectively. The differences in mean values of evaluated parameters were assessed by means of repeated-measures ANOVA with the stage of the training cycle or altitude as a repeated measure. If the results of ANOVA were statistically significant, post hoc Tukey's HSD test was applied. Mauchly's test for sphericity was included as a part of the procedure. Partial eta-squared (*η*^2^) was used as a measure of effect size. When the data did not fulfill the assumptions required for a parametric test, nonparametric Friedman's ANOVA test was used. Creatine kinase activity in athletes' serum 24 h after exercise was compared between different simulated hypoxic conditions using ANOVA and then the post hoc Tukey's HSD test. Student's *t*-test was used in the analysis of differences between lactate concentrations (La_0_ and La_max_) in serum.

Differences with a *p* value <0.05 were regarded as significant. Linear regression and Pearson's or Spearman's correlation coefficients were calculated to describe the relationships between biochemical factors and spectral fluorescence parameters of blood serum.

## 3. Results

### 3.1. Serum Fluorescence Spectra

**Figure 1:**
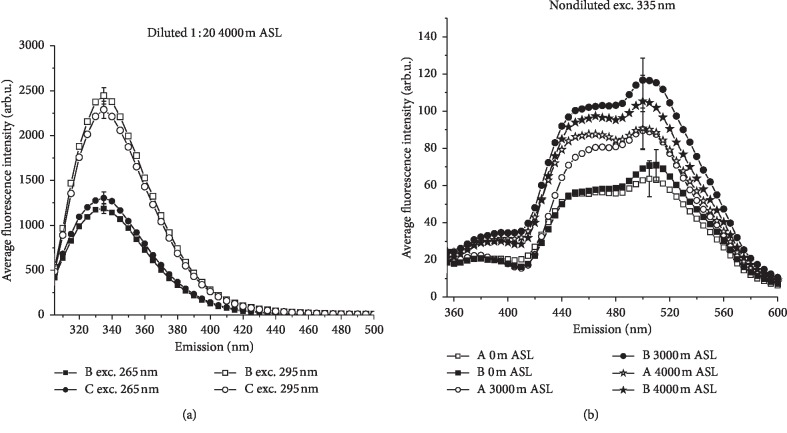
Average emission spectra of athletes' sera in normoxic and simulated hypoxic conditions for diluted samples at exc. 265 and 295 nm (a) and for undiluted samples at exc. 335 nm (b). The error bars represent the standard error of the mean (*n* = 6).

Human serum is composed of variety of components [14], but only a few of them make significant contributions to overall fluorescence. The main fluorescent species of serum have tentatively been identified by comparison with literature data as tryptophan (Trp), tyrosine (Tyr), reduced nicotinamide adenine dinucleotide (NADH), and its phosphate, pyridoxic acid lactone, pyridoxal phosphate Schiff base, and protein-bound bilirubin [36]. Their emission could be detected in two well-separated parts in the ultraviolet and visible regions (see Figures 1(a) and 1(b)), similarly as it was reported earlier for human and rat serum by Wolfbeis and Leiner [36] and Drzazga et al. [39], respectively.

Figure 1(a) displays examples of fluorescence spectra of diluted sera samples in the ultraviolet region, where tryptophan and tyrosine—two important, aromatic amino acids present in sera proteins (albumins, alpha-globulins, beta-globulins, and gamma-globulins)—emit in the same range of wavelengths. This observation can be interpreted in terms of the efficient transfer of energy from Tyr to Trp. The high emission from Trp with the maximum at 335 nm is twice as large as that for Tyr. During IHT, the fluorescence intensity of both studied amino acids was influenced by strength effort as well as high altitude.

In the visible region, markedly lower emission of metabolic compounds was observed. Fluorescence measurements for undiluted serum samples are presented in Figure 1(b). Weak broad spectra under 335 nm excitation showing the main peak at 505 nm and the second, blue-shoulder maximum at about 460 nm were found. The observed fluorescence spectra can be attributed to closely spaced overlapping emissions generated by a number of low molecular mass fluorescent compounds such as coenzymes of key enzymes in redox reactions: NAD(P)H and flavins (exc. 330–380 nm) and fatty acids (exc. 330–350 nm) [44]. Unfortunately, the boundary between different fluorescent contributions cannot be clearly drawn because of their emission bands overlapping in the visible region. The shape and amplitude of this complex spectrum can vary due to NADH(P)_bound/free_/NADH(P)_total/oxidized flavins_ ratios, depending on aerobic/anaerobic energetic metabolism and antioxidant defense as well as due to altered lipid metabolism. Moreover, its prominent shoulder in the shorter wavelength range can be assigned to the fluorescence of pyridoxic acid lactone.

It follows from Figure 1(b) that high altitudes, as well as exercise, influenced cell metabolism, causing an increase of fluorescence in the visible range. Enhancement of this emission may also be associated with the occurrence of an additional, nonenzymatic way of metabolism. It is known that the maximum emission of advanced glycation end products (AGEs) occurs at about 450–460 nm [45].

**Figure 2:**
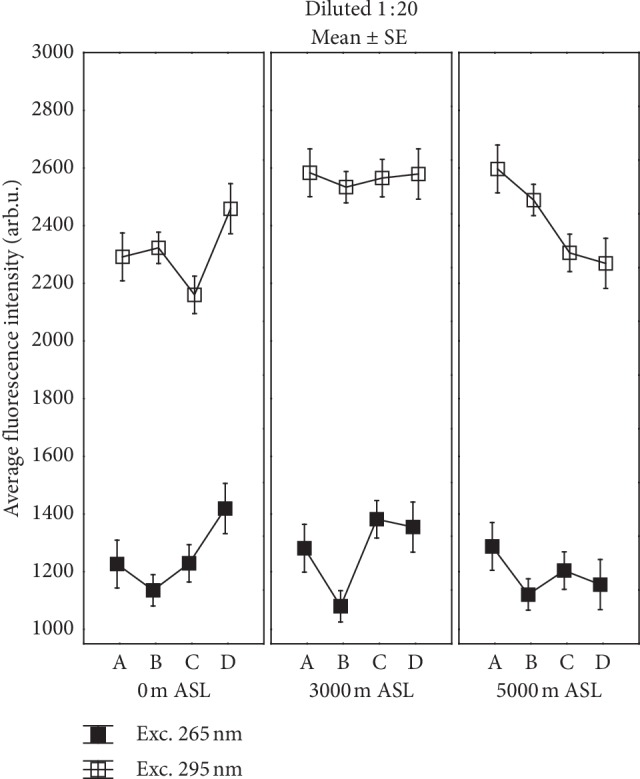
Mean values (*n* = 6) of serum amino acid (Trp and Tyr) fluorescence intensity in different times of the hypoxic training cycle: before the exercise (A), after the exercise (B), and 1 h (C) and 24 h (D) after exercise in different simulated normobaric hypoxic conditions (0, 3000, and 5000 m ASL).

**Figure 3:**
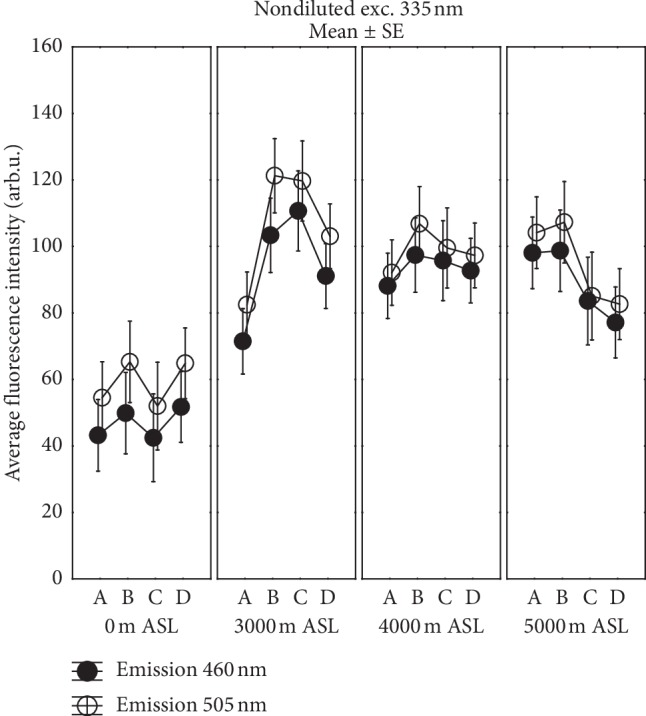
Mean values (*n* = 6) of the peak emission and blue-shoulder maximum of spectrum under exc. 335 nm for undiluted serum at different times of the training cycle (A—about 1 min before exercise, B—about 3 min after exercise, C—1 h after exercise, and D—24 h after exercise) for the studied normobaric hypoxic conditions.

Both parts of emission revealed the complex nature of autofluorescence of athletes' sera in a different way depending on normobaric hypoxic conditions and strength training. A comparison of changes in fluorescence intensity in the various stages of training for the studied normobaric hypoxic conditions is displayed in Figures 2 and 3.

Figure 2 shows the mean peak fluorescence intensity of the two main aromatic amino acids Tyr and Trp in serum collected at different time points of the hypoxic training cycle: about 1 min before exercise (A) and 3 min (B), 1 h (C), and 24 h (D) after exercise at normoxia and simulated hypoxic conditions (3000, 4000, and 5000 m ASL).

It should be noted that fluorescence of Tyr decreases after exercise (B) in all hypoxic conditions, especially markedly at 3000 m ASL, but 1 h (C) later returns nearly to its value observed before exercise (A). Results of ANOVA with the period of training cycle as a repeated measure revealed a significant impact of strength exercise. At 0 m ASL, *p*=0.001 and partial eta-squared *η*^2^ = 0.64 (as a measure of effect size), and at 3000 m ASL, *p*=0.002 and *η*^2^ = 0.62 were found. The post hoc Tukey's HSD test confirmed significant differences in fluorescence of Tyr between time points of training B and D (*p*=0.004) at 0 m ASL and at 3000 m ASL between points B and C (*p*=0.002) as well as between points B and D (*p*=0.006). Differences in the emission of Tyr disappeared at higher altitudes (4000 and 5000 m ASL) and became statistically insignificant. However, a large diversity of Tyr fluorescence response after 24 h of restitution but showing a systematic decrease with increasing altitude should be noted.

Changes in the fluorescence intensity of Trp were different from those registered for Tyr (Figure 2). Firstly, application of the simulated hypoxia conditions induced a noticeable increase of Trp fluorescence intensity in athletes' sera which was already observed before the effort, which means that the fluorescence response of Trp to deficiency of oxygen was nearly immediate. Secondly, strength exercise caused distinct changes in Trp fluorescence dependent on the altitude. At normoxia, a drop of emission 1 h after strength exercise (later than in Tyr), lack of changes at 3000 m ASL, and a marked, almost monotonic decrease of fluorescence at 5000 m ASL to the initial (A) value in normoxia were obtained. Results of ANOVA showed statistically significant changes in fluorescence under exc. 295 nm at altitude 0 m ASL (*p*=0.002 and *η*^2^ = 0.63) and at 5000 m ASL (*p*=0.002 and *η*^2^ = 0.57). Significant difference of autofluorescence of Trp was obtained between C and D time points of training (*p* < 0.001) at 0 m ASL while at 5000 m ASL between A and C (*p*=0.017) and A and D (*p*=0.075) in Tukey's test. Taking into consideration altitude as a grouping variable, Wilcoxon's paired rank test showed statistical significance between 0 and 3000 m ASL for Trp fluorescence 1 hour after exercise (*p*=0.046) and 24 hours after training (*p*=0.046) and analogically between 3000 and 4000 m ASL (in both cases *p*=0.028). Moreover, statistical difference in Trp emission between 3000 and 5000 m ASL 1 hour after training was obtained (*p*=0.028).

In addition, it is noteworthy that strength training caused interesting changes in behavior in fluorescence of both aromatic amino acids after 24 h of restitution (Figure 2). The difference in Trp fluorescence between A and D stages at 5000 ASL was greater than at other altitudes (ANOVA, *p*=0.005, partial *η*^2^ = 0.51, *p*=0.007 in Tukey's test between 5000 m ASL and 0 m ASL). A similar tendency was observed for differences in Tyr emission (*p*=0.056).

Comparison of changes in emission intensity of undiluted serum samples under exc. 335 nm in different time stages of strength training in the studied normobaric hypoxic conditions is presented in Figure 3. Considerably higher fluorescence level in the visible region was observed during training in all high altitudes, which even after 24 hours of rest did not return to that observed in normoxia. The greatest increase of emission was found for points B and C of the training cycle at 3000 m ASL. The marked drop of fluorescence at 5000 m ASL for points C and D of training in comparison with point B should be noted. Results of ANOVA with repeated measures showed a significant impact of training and high altitude on emission intensity for both analyzed maxima. ANOVA results with the altitude as repeated measure gave for the main peak *p*=0.009, partial *η*^2^ = 0.53 and *p*=0.004, partial *η*^2^ = 0.52 for the stage as repeated measure. The post hoc Tukey's HSD test indicated significant differences between 0 and 3000 m ASL after 1-hour restitution (C) (*p*=0.006) and for A and C (*p*=0.010). Significant increase in fluorescence after exercise (B) at 3000 m ASL and the following points of training A (*p*=0.008), C (*p*=0.005), and D (*p*=0.047) at 0 m ASL were also found. At 3000 m ASL, the post hoc Tukey's HSD test showed differences between A and B (*p*=0.013) as well as A and C (*p*=0.018) stages, showing a strong impact of strength effort on metabolic fluorescence at this altitude. Modification of the shape and amplitude of the blue-shoulder maximum near 460 nm were also observed. Significant increased fluorescence value of the second maximum was maintained 1 hour after exercise (C) compared to before exercise (A) (*p*=0.010) at 3000 m ASL.

### 3.2. Biochemical Assay

LDH and CK activities assayed in our experiment for four points of the training cycle at different simulated hypoxic conditions are collected in Table 1. Biochemical parameters were analyzed statistically in terms of the different stages of training as well as an oxygen deficit with increasing high altitude.

Acute hypoxia caused a little enhancement in LDH activity with altitude up to 4000 m ASL, unlike 5000 m ASL. On the other hand, strength effort increased LDH activity immediately after exercise, but during the restitution period, LDH activity returned to initial values. ANOVA results with the time points of the training cycle as repeated measure gave *p* < 0.0001 and partial *η*^2^ = 0.58. The post hoc Tukey's HSD test indicated statistically significant changes in LDH for different pairs of training stages: B and D (*p*=0.011) and B and A (*p*=0.050) in normoxia, and B and A (*p*=0.001) and B and D (*p*=0.002) at 3000 m ASL. At 4000 m ASL, significant differences (*p* < 0.03) between stages C and D, A and B, and B and D were observed. The significant changes in LDH at 5000 m ASL disappear after the effort, probably because of adaptation of the organism to hypoxic conditions.

The second parameter of muscle damage creatine kinase activity showed the very large variability among athletes in regard to stages of training cycle (Table 1), probably associated with different degrees of fitness training of sportsmen. Nevertheless, nearly a twofold decrease of creatine kinase activity at 5000 m ASL should be noted when compared to normoxia (see Table 1). ANOVA with repeated measurements conducted for CK_A_, CK_B_, CK_C_, and CK_D_ activities in 5 persons who participated in all stages of the experiment indicated a significant impact of high altitude for CK_D_ (*p*=0.026). ANOVA with the repeated time points of the training cycle showed *p*=0.0002 and partial *η*^2^ = 0.33. Tukey's HSD test pointed to a significant drop in activity of creatine kinase 24 h after training at 5000 m ASL in relation to 3000 as well as 4000 m ASL with *p*=0.043 and 0.048, respectively.

**Figure 4:**
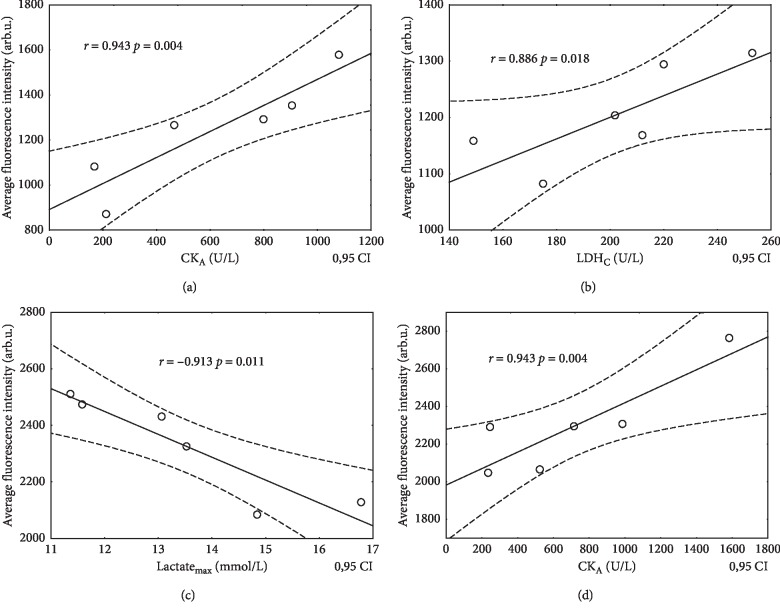
Representative correlations between the fluorescence intensity of serum fluorophores and biochemical markers. (a) Diluted 1 : 20, exc.265 nm, and altitude 4000 m ASL, about 1 min before exercise. (b) Diluted 1 : 20, exc.265 nm, and altitude 5000 m ASL, about 1 h after exercise. (c) Diluted 1 : 20, exc.295 nm, and altitude 0 m ASL, about 3 min after exercise. (d) Diluted 1 : 20, exc.295 nm, and altitude 0 m ASL, about 1 min after exercise.

In addition, large changes in lactate concentration after training dependent on hypoxic conditions were found (Figure 4). Student's *t*-test showed significant differences between LA_0_ and LA_max_ in all the studied normobaric hypoxic conditions (*p* < 0.0001 and *η*^2^ = 0.95). However, the most interesting is the marked drop of La level at 4000 and 5000 m ASL in comparison with normoxia. Results of Tukey's HSD test indicated that the LA_0_ concentration drop is statistically significant at 5000 m ASL compared to 0 m ASL (*p*=0.039) as well 3000 m ASL (*p*=0.040) while the LA_max_ decrease was significant at 5000 m ASL in relation to 3000 m ASL (*p*=0.023). A significant decrease of acidification level (above 35% in LA_0_ and 32% in LA_max_) at 5000 m ASL suggests the occurrence of the “lactic paradox” phenomenon [14, 38–46].

### 3.3. Relationships between Serum Fluorescence Intensity and Biochemical Parameters

Pearson's and Spearman's correlation coefficients were calculated to describe the relationships between biochemical blood serum markers and fluorescence spectroscopy parameters in hypoxia. Correlations were mainly obtained for the fluorescence intensity of serum amino acids with creatine kinase activity, less for La concentration and LDH activity. For 335 nm excitation, only two significant correlations of emission after exercise (B) with LA (*r* = −0.90, *p*=0.02) and LDH (*r* = 0.85, *p*=0.03) were found, in 0 m ASL and 5000 m ALS, respectively. Fluorescence intensity correlations with CK_A_ and LDH activity were positive while they were negative with LA nearly for all studied cases. Examples of the statistically significant relationships between the intensity of serum peaks under different wavelength excitations and biochemical markers obtained during IHT are presented in Figures 4(a)–4(d). Occurrence of strong correlations (*r* > 0.8) indicates that fluorescence intensity is compatible with biochemical markers routinely used in the estimation of athletes' training efficiency.

## 4. Discussion

It is interesting that the short-term stay in simulated normobaric hypoxic conditions can be reflected in the fluorescence of amino acids and metabolic compounds of serum emitting in the ultraviolet and visible regions of wavelengths, respectively. According to our knowledge, there is no information in the literature about the fluorescence of serum of athletes during IHT training. Our results showed that the emission of serum as sensitive to alterations in the function, morphology, and microenvironment in cells can provide valuable information about differences in various stages of the training in normobaric hypoxia when compared with analogical training in normoxia. The advantage of measuring serum fluorescence is the ability to obtain direct information reflecting the behavior of amino acids (Trp and Tyr) in biomolecules as well as coenzymes of key enzymes in redox reactions occurring in energetic metabolism and antioxidant defense processes related to intermittent hypoxic training. The results of fluorescent serum tests will be discussed based on recognized physiological and biochemical exercise markers taking into account the impact of physical effort and hypoxia conditions.

For Tyr, statistical changes in the fluorescence were only observed due to physical effort. The reduction of Tyr fluorescence occurred at any altitude immediately after exercise, whereas for Trp it occurred with a delay (1 hour) in normoxia while under oxygen deficiency conditions the changes in fluorescence of Trp were more complex. The largest drop (16.2%) in the emission of Tyr was observed about 3 minutes after exercise at 3000 m ASL.

Generally, the fluorescence intensity of both amino acids showed positive correlations with CK and LDH, which provides some compatibility between fluorescence intensity and biochemical markers at different altitudes and various stages of training. It is interesting that more correlations were found for CK activity than for LDH. CK molecules are smaller than LDH ones, and thus, they can show more effective and faster leakage from cells into the extracellular fluids.

The important founding is that all correlations of amino acids' fluorescence intensity with La level were negative up to 4000 m ASL but became positive at 5000 m ASL. They were statistically significant in normoxia for Tyr before exercise (*r* = −0.873, *p*=0.023) and for Trp 3 min after exercise (*r* = −0.913, *p*=0.011) while at 5000 m ASL a significant positive correlation for Trp 3 minutes after exercise was obtained (*r* = 0.835, *p*=0.038). This behavior of amino acid fluorescence in correlation with marker of acidification may suggest some conformational changes of proteins in tissues dependent on the stage of IHT experiment.

The marked differences in the differentiate plots of Trp emission in serum at the high altitudes regarding normoxia (not shown) confirmed the important role of tryptophan in our IHT experiment. The organism's desire to adapt to high-altitude effort could be particularly evident in the behavior of TRP fluorescence at an altitude of 5000 m when the value of its emission after 24 hours has approached the value before the effort in normoxia. Recently, beneficent adaptation to exercise by high-intensity interval training in hypoxia because of a significant increase concentration of HIF-1*α* and NO levels in the serum using biochemical methods was reported [47].

Moreover, the presence of free and bound tryptophan in the blood affects the process of central fatigue by forming a 5-HT neurotransmitter in the brain. The role of tryptophan, as a precursor for the synthesis of the neurotransmitter 5-hydroxyptamine (95-HT), which is involved in fatigue in different conditions of stress including endurance exercise was studied earlier [48, 49]. A significant increase in plasma 5-HT concentration in an animal model (horses) after exercise with no significantly changed concentration of Trp was found [50]. On the other hand, a significant reduction in serum concentrations of tryptophan due to exhaustive aerobic exercise was reported [51]. This study showed that changes in tryptophan-kynurenine metabolism in trained athletes are associated with increased immune activation and may influence mood and cognitive processes, which was also observed in our studies.

The amino acids tryptophan and tyrosine can be metabolized through different pathways and mediated by many factors. Our findings demonstrate clearly that Trp unlike Tyr emission is markedly influenced by normobaric hypoxic conditions. Emission of Trp is slightly higher at 3000 ASL than in normoxia and nearly does not change with the subsequent stages of training contrary to 5000 m ASL, where a progressive decrease can be seen (Figure 2). The largest statistical increase for Trp fluorescence by 13.3% was obtained before exercise at 5000 m ASL in comparison with 0 m ASL. Next Trp fluorescence at 5000 m ASL monotonically decreased with time, as if striving for the state observed in normoxia. The lowering of fluorescence of this amino acid is accompanied by a drop of lactate level after exercise. A positive relationship between the fluorescence intensity of Trp after exercise and LA_max_ content in athletes was observed (*r* = 0.835, *p*=0.038).

Lactate kinetics during endurance exercise at high altitudes (ALTs) has been considered by many groups of researchers taking into account different exercise protocols and various sets of measured parameters. Several hypotheses including metabolic adjustments by increased utilization of glucose (pyruvate) as the carbon source (acetyl-CoA) for oxidative metabolism [38], the physiological significance of hypoxia-induced reactive oxygen species (ROS) formation and their role in stabilization of HIF-1*α* in a low O_2_ environment [52], and synthesis alternation of the synergy between protein synthesis and degradation leading to muscle atrophy [22] have been proposed to explain the “lactate paradox.”

The pronounced changes in emission in the visible region (see Figure 3) observed in our research prove that IHT based on exhaustive exercise affects cell energy metabolism and protein metabolism in human skeletal muscle, as was noted earlier [53]. The highest intensity of complex fluorescence under exc. 335 nm was observed at 3000 m ASL and it did not return to the normoxia-specific value even after 24 hours of rest. However, a striving for fluorescence intensity lowering to the normoxia-specific value was noticeably evident for training at 5000 m ASL. However, in the visible range, the fluorescence deviations from the status observed during exercise in normoxia for hypoxia cases should be considered qualitatively. Emission intensity is low and results from the overlap of spectra of several different fluorescent components (NAD(P)H, flavin, and fatty acids), often dependent on each other in energy metabolism and antioxidant defense processes. Using them as markers in autofluorescence would require refinement of research using the characteristic wavelengths of their excitation in subsequent studies.

On the other hand, an intriguing finding at 5000 m ASL was nearly a twofold drop of creatine kinase activity in comparison with normoxia (or 3000 m ASL). According to our knowledge, such large changes of CK during training at 5000 m ASL were not published earlier. Decrease of this biomarker value, e.g., from CK_A_ (3000 m ASL) = 661 ± 159 to CK_A_ (5000 m ASL) = 339 ± 77 or from CK_D_ (3000 m ASL) = 902 ± 195 to CK_D_ (5000 m ASL) = 535 ± 133) seems to be associated with sealing the cell membrane, e.g., due to stress, free radicals remain up to 24 h after exercise. However, the response of LDH—the other indicator of cell damage—was distinctly weaker. LDH activity was only slightly influenced by high altitude, unlike CK.

It follows from our analysis that just being in normobaric hypoxic conditions changed the fluorescence properties of the serum. An increase of fluorescence intensity was found in both studied ranges of emission. Significant changes in serum fluorescence and relatively weak but adverse modifications in biochemical markers (an increase of La, CK, and LDH) occurred at 3000 m ASL, and next, they decreased slightly at 4000 m ASL. At 5000 m ASL, fluorescence values were clearly changing in the direction of those observed in normoxia and improvement of biological markers, i.e., a marked drop of La and CK, was observed. Such modifications in fluorescence and biochemical assay suggest an increase of adaptation of the organism to the effort in deficiency of oxygen conditions at 5000 m ASL unlike 3000 m ASL.

We hope that our new results of serum autofluorescence associated with biochemical assay will contribute to the understanding of metabolic regulation and physiological response to IHT in normobaric hypoxic conditions. In addition, they may be helpful in the planning of suitable training in high altitude to improve sports performance in elite athletes.

## 5. Study Limitations

To the best of our knowledge, our study is the first in which the results of serum autofluorescence and biochemical markers in athlete's response to strength effort were compared in hypoxia and normoxia. Our investigations, however, are not without certain limitations. Firstly, we conducted study on a small sample size. This is due to the fact that during the recruitment of volunteers, after explained the possible risk of performing in the study, some candidates resigned from participation in the study. Next limitation is the lack of information whether the results are comparable in other groups with different fitness level and age. Future studies should include this aspect.

## 6. Conclusions

Autofluorescence studies of athletes' serum can provide new information on the role of aromatic amino acids present in proteins and cell metabolism during intermittent hypoxic training in simulated hypoxic conditions. Fluorescence intensity of Tyr seems to be more sensitive to strength effort whereas Trp emission is more influenced by a deficiency of oxygen. We suggest that endogenous aromatic amino acids, especially tryptophan, could be exploited as intrinsic biomarker in autofluorescence studies. The pronounced changes in the visible range of fluorescence of key enzymes in redox reactions and accumulated lipids due to high altitudes prove that cell energy metabolism in trained athletes is dependent on normobaric hypoxic conditions. Especially substantial changes in biochemical and spectral parameters were found at 5000 m ASL. The significant decrease of lactate level (lactic paradox) and nearly twofold drop of creatine kinase activity in comparison with normoxia as well as pronounced changes in serum emission in the restitution period in the direction towards values distinctive for normoxia indicate adaptation of the organism to the effort in the deficiency of oxygen conditions. Analysis of serum fluorescence accompanied by biochemical assay may be helpful in the planning of suitable intermittent hypoxic training to improve sports performance of athletes.

Furthermore, the study demonstrated a beneficial effect of the analysis of serum autofluorescence in the assessment of athlete's response to effort in hypoxia. These studies confirm the usefulness of this method to *monitoring the athlete's training response* to acute and chronic hypoxia. However, the results from various research groups suggested individual variability in response to hypoxia. It is worth noting that, in our results of serum autofluorescence, we also observed relatively high individual variability in response. Regardless of attempts to find an effective tool to analyze individual response to acute and chronic hypoxia, results of serum autofluorescence during altitude training may be a probably useful method for identification of responders and nonresponders prior to altitude. These and many other issues require further research.

## Figures and Tables

**Table 1:** Mean values ± SEM of LDH (U/L) and CK (U/L) at four time points of the training cycle in different hypoxia conditions.

Biochemical parameters	Mean values ± SEM (*n* = 6)			
	0 m ASL	3000 m ASL	4000 m ASL	5000 m ASL
LDH_A_ (U/L)	183 ± 13	192 ± 10	196 ± 7	178 ± 5
LDH_B_ (U/L)	220 ± 9	238 ± 11	232 ± 6	205 ± 8
LDH_C_ (U/L)	191 ± 11	204 ± 10	223 ± 14	202 ± 18
LDH_D_ (U/L)	177 ± 11	193 ± 11	186 ± 11	188 ± 12
CK_A_ (U/L)	652 ± 219	661 ± 159	605 ± 155	339 ± 77
CK_B_ (U/L)	822 ± 285	831 ± 199	732 ± 186	416 ± 93
CK_C_ (U/L)	758 ± 241	793 ± 187	726 ± 170	430 ± 97
CK_D_ (U/L)	950 ± 360	902 ± 195	887 ± 182	535 ± 133
Lactate_0_ (mmol/L)	1.81 ± 0.24	1.81 ± 0.23	1.47 ± 0.22	1.17 ± 0.08
Lactate_max_ (mmol/L)	13.52 ± 0.84	14.54 ± 1.42	12.58 ± 1.34	9.83 ± 0.98

## Data Availability

The data used to support the findings of this study are available from the corresponding author upon request.
